# Effects of Ultrasound Modification with Different Frequency Modes on the Structure, Chain Conformation, and Immune Activity of Polysaccharides from *Lentinus edodes*

**DOI:** 10.3390/foods11162470

**Published:** 2022-08-16

**Authors:** Yu Hua, Haihui Zhang, Qian Fu, Yuqin Feng, Yuqing Duan, Haile Ma

**Affiliations:** 1 School of Food and Biological Engineering, Jiangsu University, Zhenjiang 212013, China; 2 Institute of Food Physical Processing, Jiangsu University, Zhenjiang 212013, China

**Keywords:** lentinan, ultrasonic frequency, chain conformation, triple-helix, structure–activity relationship, immune activity

## Abstract

**Highlights:**

- Effects of ultrasonic frequency modes on the lentinan conformation were studied.
- Ultrasonic treatment could transform triple-helix chain into single helix chains.
- Dual-frequency ultrasound can change the triple-helix chain from rigid to flexible.
- Dual-frequency ultrasound significantly improved the immune activity of lentinan.

**Abstract:**

The aim of this study was to investigate the effects of ultrasound with different frequency modes on the chemical structure, chain conformation, and immune activity of lentinan from *Lentinus edodes*; the structure–activity relationship of lentinan was also discussed. The results showed that, compared with original lentinan (extracted using hot water), although ultrasonic treatment did not change the monosaccharide composition and main functional groups of lentinan, it significantly changed its chain conformation. Especially at 60, 40/60, and 20/40/60 kHz, according to atomic force microscopy and solution chain conformation parameters, lentinan transformed from a rigid triple-helix chain to a flexible single-helix chain, and the side-chain was severely broken. Under this condition, lentinan had the worst immune activity. However, at 20/40 and 20/60 kHz, the rigid triple-helix chain transformed into a loose and flexible triple-helix chain, showing the strongest immune activity. These results indicated that dual-frequency ultrasound had significant effects on the conformation of lentinan, and the conformation characteristics of polysaccharide chain such as spiral number, stiffness and flexibility, and side-chain played an important role in immune activity. This study shows the great potential of ultrasound with different frequency modes in carbohydrate food processing, which may have important reference value and practical significance.

## 1. Introduction

As a well-known medicinal and edible mushroom, *Lentinus edodes* has been cultivated in China, Japan, South Korea, and Russia for more than 2000 years [1]. *L. edodes* contains a variety of nutrients, such as carbohydrates (58–60%), protein (20–23%), fiber (9–10%), lipids (3–4%), and ashes (4–5%) [2]. Lentinan is considered to be the most important bioactive macromolecule in *L. edodes* [3], and its main active component β-(1-3)-glucan has a variety of bioactivities, including immunomodulatory [4], antioxidant [5], antitumor [6], and antiviral [7] properties, which may be potentially applied in food, cosmetics, and medicines.

Most studies have shown that the biological activity of polysaccharides is closely related to their chemical structure [8], molecular weight [9], and chain conformation. In recent years, polysaccharides with a triple-helix conformation have attracted extensive attention because of their outstanding antitumor [10] and immunomodulatory activities [11]. Lentinan is a typical polysaccharide with triple-helical conformation [12]. Existing data show that the conformation of lentinan could be transformed from a triple helix to single random coil in dimethyl sulfoxide (DMSO) [13] or NaOH aqueous solution [14], or at 140 °C [15]. This is mainly due to the breaking of hydrogen bonds between the triple-helical chains of lentinan. When the triple helix is broken into a single random coil, its antitumor activity and immunomodulatory activity are significantly reduced or disappear [16]. Therefore, it is of great significance to clarify the changes of chemical structure, chain conformation, and biological activity of lentinan for the successful application of lentinan in disease prevention and treatment. 

In recent years, due to the great potential in the degradation and modification of active polysaccharides, ultrasound has become a good auxiliary tool for the study of structure–activity relationship of polysaccharides [17]. As an environmentally friendly, efficient, and low-cost physical processing method, ultrasound has been widely used for the extraction of various polysaccharides [18,19]. More attractively, ultrasound can alter the spatial structure of polysaccharides by destroying intermolecular and intramolecular hydrogen bonds, thereby changing their biological activities [20], such as enhancing antioxidant and immune activities [21,22]. The modification of polysaccharide using ultrasound results from the rupture of glycosidic bonds, which is closely related to the high shear force caused by the rapid collapse of cavitation bubbles. After ultrasonic irradiation, the weight average molecular weight (M_w_) of carboxylic curdlan decreased obviously, and the mean radius of rotation (R_g_) increased first and then decreased. Carboxylic curdlan molecules existed in a more flexible and extended random curl conformation in aqueous solution [23]. Under the action of single-frequency ultrasonic, the change in ultrasonic frequency will cause different cavitation effects. Chen et al. [24] found that the molecular weight of pectin decreased from 8.43 × 10^4^% ± 0.21% Da to 7.20 × 10^4^% ± 0.22% Da as the ultrasonic frequency increased from 20 kHz to 40 kHz, and pectin treated with ultrasound at 40 kHz showed less aggregation or looser networks. Studies have shown that the number of bubbles formed using multifrequency ultrasound is more than five times that formed using single-frequency ultrasound [25], resulting in a much higher cavitation effect [26] and more effective extraction of bioactive components. Furthermore, compared with the single-frequency ultrasonic reactor, the multifrequency ultrasonic reactor has a variety of waves, which provides a wider frequency range, and the addition of mechanical interference and a cavitation core can overcome the inhomogeneity of energy dissipation and the limited extraction yield in the reaction medium. For example, Chen et al. [27] found that ultrasonic extraction at different frequencies resulted in significant changes in the extraction rate, molecular weight (M_w_), surface morphology, and rheological properties of okra polysaccharides. At 40/60 kHz, the okra polysaccharides with the lowest M_w_ (0.85–14.93 × 10^5^ Da), the highest polyphenol content (7.38%), and the loosest network structure showed better antioxidant activity, cholesterol absorption, and nitrous ion absorption than others. Yang et al. [28] found that the maximum extraction rate of *Hovenia dulcis* polysaccharides under dual-frequency extraction was 9.02 ± 0.29%. Compared with single-frequency and triple-frequency extraction, *Hovenia dulcis* polysaccharides under dual-frequency extraction also showed the best oil holding ability, foaming ability, and emulsifying ability. The antioxidant activity of *Hovenia dulcis* polysaccharides under dual-frequency extraction was better than that under single-frequency and triple-frequency extraction. These results indicate that the generation and intensity of ultrasonic cavitation are strongly influenced by ultrasonic frequency, which largely determines the cavitation rate in solution [29], and then affects the chemical structure and chain conformation of polysaccharides. Up to present, the extraction of polysaccharides is still based on hot water or acid/alkali-base water. The low dissolution rate and poor solubility of polysaccharides often lead to a significant reduction in their biological activities. Ultrasonic assisted extraction has been used in the processing and preparation of edible fungi and plant polysaccharides, and it is significantly superior to the traditional hot water method in terms of extraction efficiency and improving the biological activity of polysaccharides, showing unique advantages and good application prospects. However, the current research still focuses on the optimization of process parameters, the molecular structure and biological activity of polysaccharides and other macro information, while the molecular structure, chain conformation transition, and biological activity of polysaccharides, as well as their relationships, are still unclear and need to be further studied.

Therefore, in this study, lentinan was modified using single-frequency, dual-frequency, and triple-frequency ultrasound. The effects of different frequencies and combinations of ultrasound on the chemical structure and chain conformation of lentinan were studied, the changes in immune activity were evaluated, and the conformation relationship between lentinan structure and immune activity was discussed. These studies may lay a theoretical foundation for the process control of frequency ultrasonic processing and screening of active polysaccharides.

## 2. Materials and Methods

### 2.1. Materials and Equipment

*L. edodes* was purchased from Hubei Academy of Agricultural Sciences (Wuhan, China). Monosaccharide standards (d-mannose, l-rhamnose, d-galacturonic, d-glucose, d-galactose, and l-arabinose), sulfuric acid, Congo red, 1,1-diphenyl1-2-picryl hydeazyl (DPPH), 2,2′-azino-bis (3-ethylbenzo-thiazoline-6-sulfonic acid) (ABTS), and dimethyl sulfoxide (DMSO) were purchased from Sigma-Aldrich Chemical Co. (St. Louis, MO, USA). All other chemical reagents were analytical reagent grade.

Lab-scale multifrequency power ultrasound equipment designed by Jiangsu University was used for the experiments. It was equipped with three different frequency generators (20, 40, and 60 kHz), and the maximum output acoustic power of each generator was 300 W. There were three working modes: single-frequency, dual-frequency, and triple-frequency ultrasound. Details are shown in Supplementary Figure S1.

### 2.2. Preparation and Ultrasonic Treatment of Lentinan

Pretreatment of sample: The dried fruiting bodies of *L. edodes* (200 g) were ground and sieved through 40 mesh, and the lipids were removed with petroleum ether. Then, the *L. edodes* was dried in an oven at 37 °C, collected, and stored at −20 °C for further analysis.

Preparation of lentinan: *L. edodes* was mixed with deionized water (1:20 g/mL), extracted twice with hot deionized water at 90 °C for 3 h, and subjected to the Sevag method (*n*-butanol alcohol to chloroform 1:4) five times to remove free proteins. Then, 95% (*v*/*v*) alcohol was added to the resulting solution slowly, stirring until the concentration of the alcohol reached 75%. The mixtures were centrifuged (5000 rpm, 15 min); then, the precipitates were collected and intensively dialyzed for 72 h against ultrapure water (cutoff M_w_ 3500 Da) to remove the small-molecular compounds (e.g., flavonoids or polyphenols). After concentration and freeze-drying, the lentinan was extracted with multifrequency power ultrasound (MFPU) in the following modes: single frequency (20, 40, and 60 kHz), dual frequency (20/40, 20/60, and 40/60 kHz), and triple frequency (20/40/60 kHz). The samples were subjected to ultrasonication treatment for 45 min with 15 s on and 2 s off with the power fixed at 300 W. The ultrasonic temperature (25 ± 1 °C) was kept constant with water bath circulation. After ultrasonic treatment, the lentinan was lyophilized and stored at 4 °C for further analysis. Lentinan without ultrasonic treatment was used as the control sample.

### 2.3. Characterization of Polysaccharides 

#### 2.3.1. UV–Visible (UV–Vis) Spectroscopy

The UV absorbance of lentinan solution (1 mg/mL) treated with different ultrasound modes was determined on a UV/visible spectrophotometer (Carry-100, Varian, Palo Alto, CA, USA) in the 200–400 nm wavelength range.

#### 2.3.2. Monosaccharide Composition Analysis

The monosaccharide composition of lentinan was analyzed using gas chromatography (GC) according to a previously reported method with minor modification [30]. Briefly, lentinan (10 mg) was hydrolyzed with 4 mL of 2 M trifluoroacetic acid (TFA) at 110 °C for 8 h to obtain a polysaccharide hydrolysate. Subsequently, the polysaccharide hydrolysate was treated with 10 mg of hydroxylamine hydrochloride and pyridine (1.0 mL) at 90 °C for 30 min and cooled, before adding acetic anhydride; it was then subjected to acetylation to 90 °C for 30 min, and finally filtered through a 0.22 μm organic phase filter membrane. The derivative was analyzed using a gas chromatograph (7890 A, Agilent Technologies, Palo Alto, CA, USA).

#### 2.3.3. FT-IR Spectroscopy Analysis

The monosaccharide types, glycosidic bonds, and functional groups of lentinan were analyzed using FT-IR according to a previously described method with minor modification [31]. The samples were ground in an agate mortar with dried KBr (1:100 mg) and pressed into a 1 mm pellet for FT-IR analysis (a Nicolet is50 FT-IR Spectrometer, Thermo Electron, Madison, WI, USA) at a frequency range of 4000–400 cm^−1^.

#### 2.3.4. Circular Dichroism (CD) Spectra

Lentinan (0.1 mg/mL) was placed in a quartz sample vessel with a diameter length of 0.1 cm. CD spectra of different lentinans were obtained in the 190–250 nm wavelength range using a circular dichroism chromatograph (JASCO J-815, Tokyo, Japan) at room temperature.

#### 2.3.5. Scanning Electronic Microscopy (SEM)

The dried lentinan powder was uniformly fixed on the sample table with conductive adhesive, and the surface was coated with a layer of 3–30 nm gold film. The surface morphology of lentinan was observed with an SEM (S-3400 N, Hitachi, Japan).

#### 2.3.6. Congo Red Analysis

The Congo red method was used to determine whether lentinan contains a triple-helix conformation [32]. The lentinan (1.0 mg/mL) was fully mixed with Congo red solution (91 μmol/L), and 1.0 mol/L NaOH was added to the mixture dropwise until the concentration was within the range of 0–0.5 mol/L. The maximum absorbance was measured using a UV/vis spectrophotometer (Implen Nanophotometer, Munich, Germany) in the range of 400–600 nm. 

#### 2.3.7. Analysis of Molecular Weight and Chain Conformation

The determination of molecular weight was modified slightly according to the method reported in the literature [23]. The weight-average molecular weight (M_w_), number-average molecular weight (M_n_), molecular weight distribution (M_w_/M_n_), *z*-average radius of gyration (R_g_), and chain conformation parameters of lentinan were determined using size-exclusion chromatography coupled with multiangle laser light scattering (SEC-MALLS, DAWN HELEOS II, Wyatt Technology Co., Santa Barbara, CA, USA). OHpak SB-806 M HQ and SB-805 HQ gel chromatographic columns were connected in series, and OHpak SB-G was used as a guard column; the column temperature was 25 °C, the flow rate was 0.5 mL/min, the sample injection volume was 200 μL, and the refractive index increment was 0.138 mL/g. The online Astra software (version 6.1.7, Wyatt Technologies, USA) attached to the MALLS system was employed for data collection, processing, and analysis. The hydrodynamic radius (R_h_) of lentinan was measured using DLS.

#### 2.3.8. Intrinsic Viscosity Measurements

The intrinsic viscosity ([η]) of lentinan was measured using a glass Ubbelohde-type capillary viscometer (0.5–0.6 mm capillary diameter) in a thermostatic water bath at 30 ± 0.1 °C. The kinetic energy correction was negligible. The value of [η] was determined using the following Solomon–Ciutâ equation:

η_r_ = t_s_/t_0_, η_sp_ = η_r_ − 1,

$$[\eta ]=\frac{1}{c}\sqrt{2\left(\eta_{\mathrm{sp}}-{\ln\eta }_{r}\right)},$$

where t_s_ and t_0_ are the flow times of the lentinan solution and the solvent, respectively, c represents the concentration of lentinan (g/mL), η_sp_ represents the specific viscosity, and η_r_ represents the relative viscosity. 

#### 2.3.9. Atomic Force Microscopy Analysis

The morphology of lentinan was observed using atomic force microscopy (AFM, Bruker, Brerica, MA, USA) [5]. Briefly, 5–10 μL of lentinan solution (10 μg/mL) was dropped onto mica flakes with a fresh and flat surface, left to dry completely, and then observed using AMF. The Nanoscope software was used for all image analysis and processing. 

#### 2.3.10. X-ray Diffraction Spectroscopy

The crystallization properties of lentinan before and after ultrasonic treatment were determined using an X-ray diffractometer (Rigaku Inc., Tokyo, Japan) with Cu Kα radiation. The operating conditions were as follows: the voltage and current were 40 kV and 40 mA, scanning range was 5–80°, and scanning rate was 4°/min. 

### 2.4. In Vitro Immunomodulatory Activity Assay

#### 2.4.1. Cell Culture

The RAW 264.7 cells were grown in complete medium (DMEM) supplemented with 10% FBS (*v*/*v*), and 1% (*v*/*v*) penicillin/streptomycin. Then, samples were incubated at 37 °C in a humidified atmosphere of 95% air and 5% CO_2_.

#### 2.4.2. Cell Proliferation Assay

The effect of lentinan on cell proliferation was determined using Cell Counting Kit-8 (CCK-8). Briefly, the RAW264.7 cells at logarithmic growth stage were adjusted to 1 × 10^5^ cells/mL and inoculated at 100 μL per well into 96-well plates. The cells were incubated at 37 °C and 5% CO_2_ for 24 h; then, the supernatant was removed, and the pellet was washed with PBS three times. The experimental group included a blank control group (100 μL of complete medium, DMEM), positive control group (100 μL of LPS with concentration of 1 μg/mL), and a sample group (100 μL of lentinan solution with final concentrations of 50μg/mL, 100 μg/mL, 200 μg/mL, 400 μg/mL, and 800 μg/mL, respectively). Six wells were set for each group. After 12 h culture in a 5% CO_2_ cell incubator at 37 °C, the supernatant was discarded, the pellet was washed with PBS three times, and 100 μL of 10% CCK8 solution was added. The experimental group was incubated in a 5% CO_2_ cell incubator at 37 °C for 1–2 h. Lastly, the absorbance value was measured at 450 nm with a microplate reader. The cell viability was calculated according to the following equation:

Cell viability% = A_sample_/A_blank_ × 100%.

#### 2.4.3. Phagocytosis Assay

The effect of lentinan on the phagocytic activity of RAW264.7 cells was determined using the neutral red method. Macrophages were incubated in 96-well plates for 12 h, and then cultured with 200 μg/mL of lentinan samples for 24 h. After culture, the supernatant was discarded, and 100 μL of 0.075% neutral red normal saline was added to each well, before culturing in the incubator for 2 h. Then, the supernatant was removed, and the pellet was washed with PBS three times. Afterward, 100 μL of cell lysis solution (acetic acid/ethanol (1 M) = 1:1) was injected into each well, before culturing at 25 °C for 1–2 h. Lastly, the absorbance value was measured at 550 nm. The cell phagocytic activity was calculated using the following formula:

Phagocytic activity% = A_sample_/A_blank_ × 100%.

#### 2.4.4. Cell Morphological Observation

The morphology of macrophages was observed under a inverted fluorescence microscope, including a blank control group (100 μL of complete medium, DMEM), positive control group (100 μL of LPS with concentration of 1 μg/mL), and sample group (100 μL of lentinan solution before and after different frequencies and combinations of ultrasound with final concentration of 200 μg/mL). 

#### 2.4.5. Determination of Nitric Oxide (NO)

NO was determined according to the instructions of kit. Briefly, after culture, 50 μL of supernatant was collected, Griess Reagent I and Griess Reagent II were successively added, and the sample was shaken evenly. The absorbance of the mixture at 540 nm was measured using a microplate reader, and NaNO_2_ was used to prepare a standard curve.

#### 2.4.6. Measurement of Immune Factors

The cell density and dosimetry of each well were the same as above, and the cells were inoculated into 24-well plates with 500 μL per well. After culture, the supernatant was collected and centrifuged at 1500 rpm for 5 min. TNF-α, IL-6, and IL-1β contents were determined using ELISA.

## 3. Results and Discussion

### 3.1. Chemical Structure Characterization

#### 3.1.1. UV Spectral Analysis

**Figure 1:**
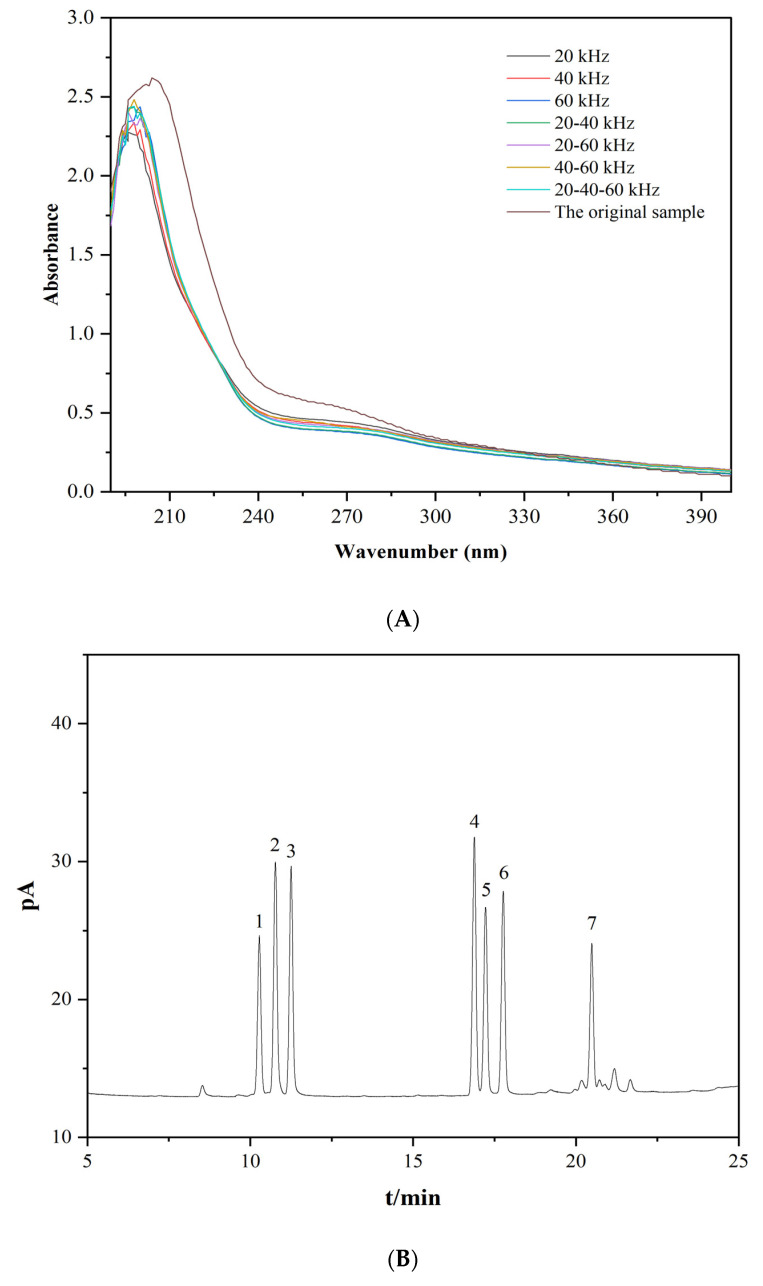

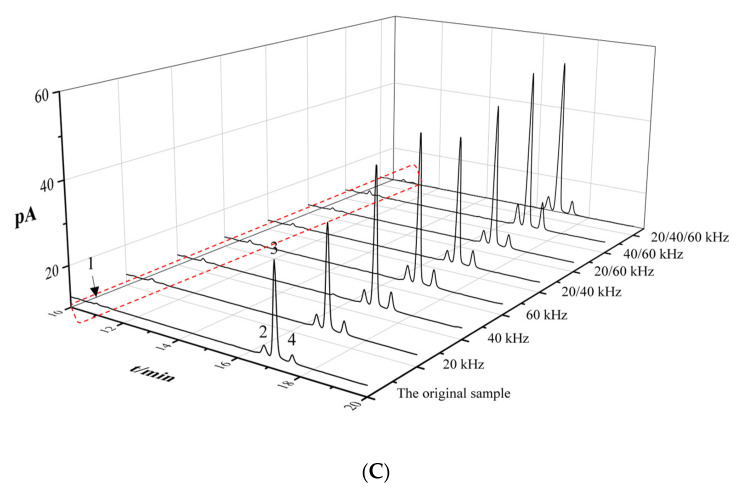
UV spectrum of lentinan (**A**); gas chromatogram of a standard monosaccharide saccharin acetate derivative: (1) rhamnose, (2) arabinose, (3) xylose, (4) mannose, (5) glucose, (6) galactose, (7) inose (interior label) (**B**); monosaccharide composition of lentinan (**C**).

As shown in Figure 1A, lentinans treated using ultrasound with different frequency modes showed a very weak absorption peak at about 280 nm, indicating that these lentinans may contain a small amount of binding proteins, because the free proteins were basically cleared by Sevag reagent [33]. In addition, the UV spectrum of lentinan at different frequencies had no absorption peak at about 260 nm, indicating that these polysaccharides did not contain nucleic acid [34].

#### 3.1.2. Monosaccharide Composition Analysis

As shown in Figure 1C, ultrasound with different frequency modes did not change the monosaccharide composition of lentinan, but changed the molar percentage of monosaccharides (Table 1), such as Glu, Ara, Gal, and Man, consistent with previous reports [35]. Whether single-frequency, double-frequency, or triple-frequency ultrasound, the molar ratio of Glu increased, while the molar ratio of Gal and Man decreased, which may be because the side-chains (Ara, Gal, and Man) of lentinan are more easily broken under the action of ultrasound. As the main backbone structure, Glu is not easily damaged; hence, the molar ratio of glucose increased [24]. Moreover, ultrasound can change the arrangement of hydrogen and hydroxyl groups on carbon atoms with interconversion between individual monosaccharides, resulting in the conversion of side-chain monosaccharides to backbone monosaccharides [36]. In particular, triple-frequency ultrasonic (20/40/60 kHz) had the largest influence on the molar ratio of monosaccharide, whereby the molar ratio of Glu increased from 77.51% to 81.53%. This made the ultrasound effect more pronounced as the superposition of ultrasound frequencies may have had positive effects on cavitation, as well as thermal and mechanical effects.

#### 3.1.3. FT-IR Analysis

**Figure 2:**
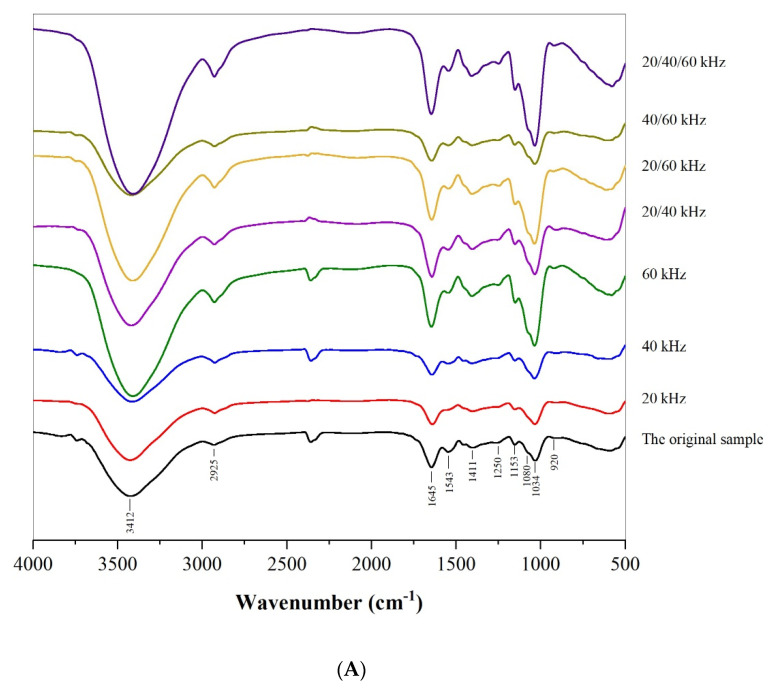

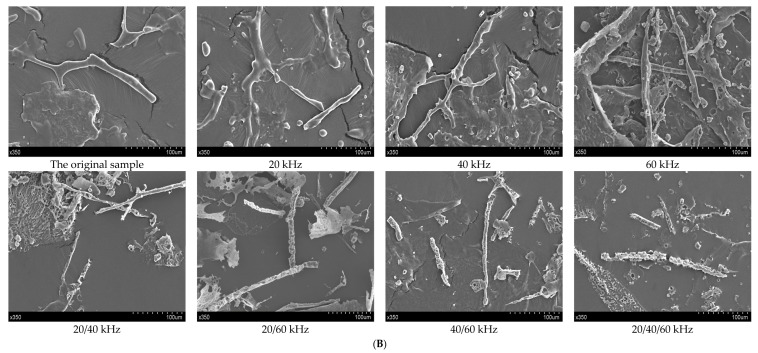
FT−IR spectra of lentinan in the range of 4000−500 cm^−1^ (**A**); SEM images (350× magnification, scale bar 100 μm) of lentinan (**B**).

The characteristic absorptions of lentinan treated using ultrasound at different frequencies were analyzed according to the FT-IR spectra in the range of 400–4000 cm^−1^. As shown in Figure 2A, the functional groups on the polysaccharide chains were essentially the same. Obviously, the strong and broad absorption peak around 3412 cm^−1^ and weak signal around 2925 cm^−1^ were attributed to stretching vibration of –OH (3200–3600 cm^−1^) and C–H resonance of methyl group in sugar ring (2800–3000 cm^−1^), respectively. These absorption peaks represent characteristic groups of polysaccharides [37]. The absorption peaks at 1645 cm^−1^ and 1543 cm^−1^ were the crystal water of polysaccharide (1600–1650 cm^−1^) or variable angle vibration of primary amino acid N–H (1500–1650 cm^−1^), which indicated that crystal water or bound protein may be present in the polysaccharide [38]. The absorption peak at 1411 cm^−1^ was due to C–C stretching vibration. The absorption peak at 1250 cm^−1^ was the stretching vibration of O=S=O of polysaccharide (1200–1400 cm^−1^). Moreover, the absorption at 1153 cm^−1^, 1080 cm^−1^, and 1034 cm^−1^ was ascribed to stretching vibrations of the C–O–H pendant group, as well as C–O–C glycosidic band vibrations of the pyranose ring and expansive vibrations of the asymmetric ring. The absorption peak at 920 cm^−1^ represented the vibration of β-type glycosidic linkages [39]. Therefore, these compounds were carbohydrates, containing β-glycosidic bonds, but not α-glycosidic bonds, mainly with a pyranose ring structure. In conclusion, the results obtained from FT-IR analysis indicated that ultrasound with different frequency modes could lead to the breaking of polysaccharide glycosidic linkages without a significant effect on the main functional groups of polysaccharides. 

#### 3.1.4. SEM Analysis

Scanning electron microscopy (SEM) was used to intuitively evaluate the changes in microstructures of lentinan before and after ultrasound treatment. As depicted in Figure 2B, compared with the original lentinan, the surface of lentinan treated using ultrasound became rough and exhibited significant differences in size, shape, and porosity due to different frequency modes. Among them, triple-frequency ultrasound (20/40/60 kHz) showed the strongest impact on the morphology of lentinan, followed by dual-(40–60 kHz) and single-frequency ultrasound (60 kHz). After ultrasonic treatment, the surface of lentinan became rough, with more pores and fragments, which may have been the result of a large number of ultrasonic cavitation activities, turbulent shear, and instantaneous high pressure [40], while the superposition of ultrasonic frequencies greatly intensified the ultrasonic cavitation, turbulent shear, and instantaneous high pressure, further leading to the splitting of polysaccharides into thin and small fragments with a loose network shape [41]. 

### 3.2. Chain Conformation

#### 3.2.1. Congo Red Test

**Figure 3:**
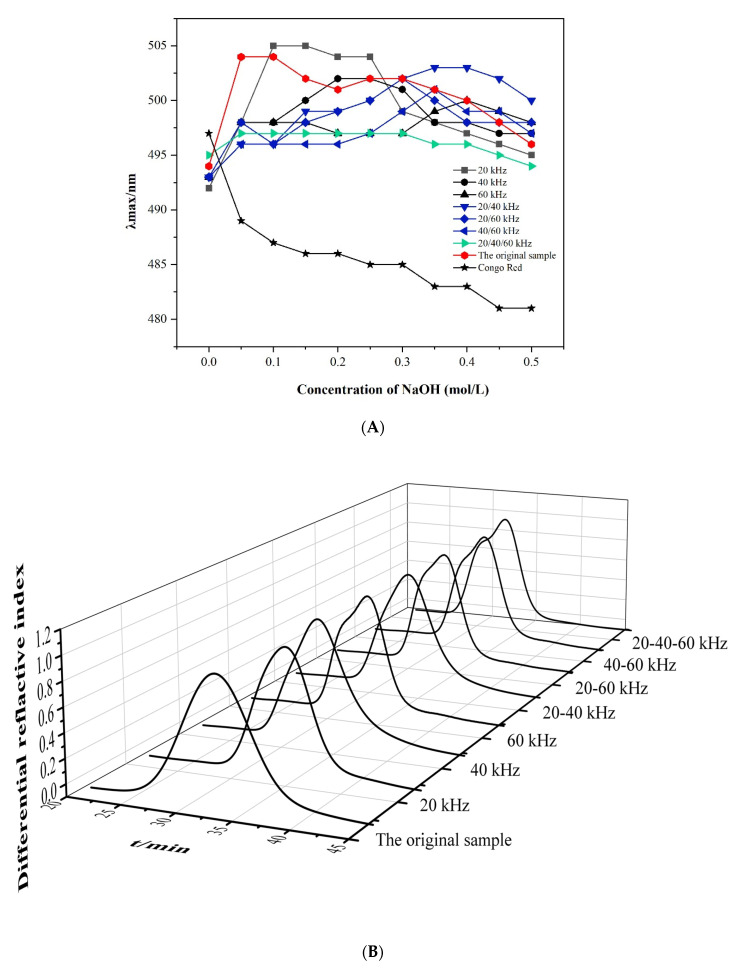

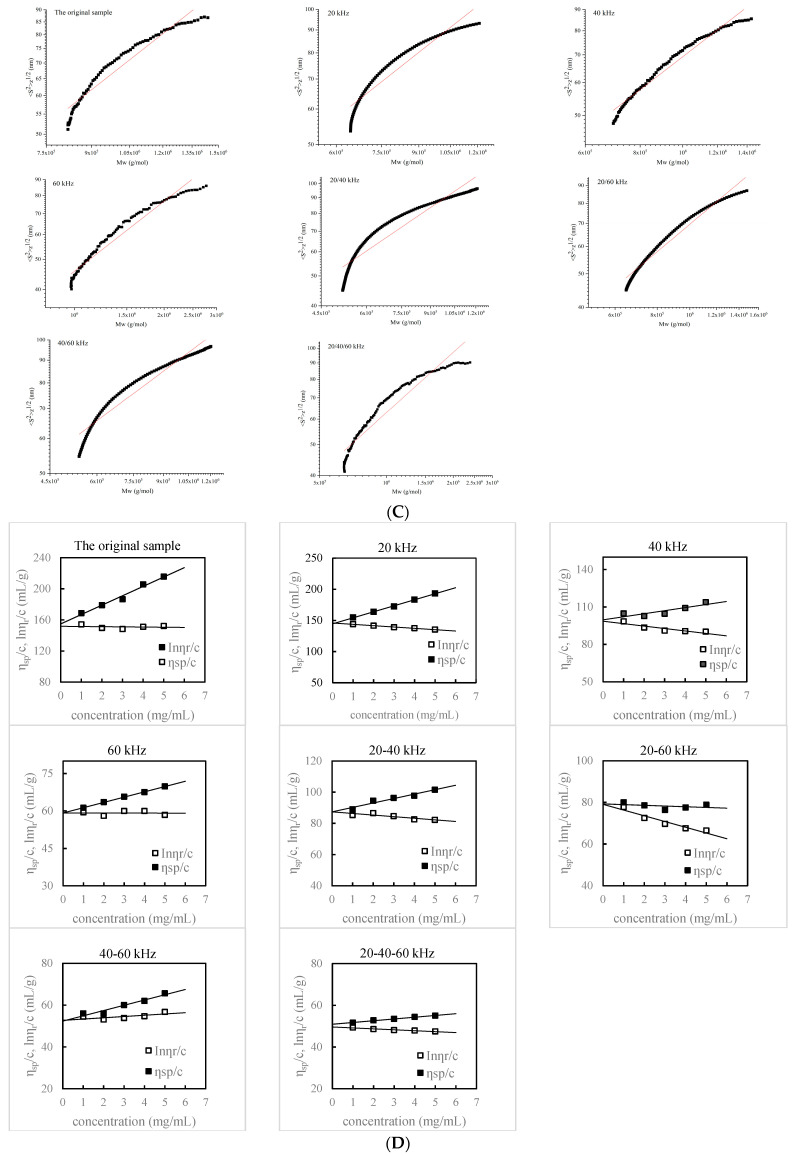
Changes in absorption maximum (λmax) of Congo red for ultrasound-treated curdlans at various NaOH concentrations (**A**); HPSCE chromatograms of lentinan (**B**); logarithmic plot of lentinan M_w_ versus R_g_ (**C**); dependence of η_sp_/c and lnη_r_/c on concentration c (**D**).

Polysaccharides containing a triple-helix conformation can interact with Congo red and form a Congo red–polysaccharide complex, resulting in a red shift of the maximum absorbed wave (λ_max_) [42]. A decline in the red shift of the Congo red–polysaccharide complex can be observed when the triple-helical conformation is destroyed by strong alkaline. Therefore, the changes in the triple-helix structure of lentinan could be evaluated by detecting λ_max_ before and after ultrasound treatment. As shown in Figure 3A, the original lentinan showed an obvious red shift (from 492 nm to 504 nm), indicating the presence of a typical ordered triple-helix conformation [43]. However, for lentinan treated using ultrasound, the values of λ_max_ varied greatly due to different ultrasonic frequency modes. When the frequencies were 60 kHz, 40/60 kHz, and 20/40/60 kHz, there was no obvious red shift after ultrasonic treatment, indicating that the triple helix in polysaccharides was destroyed, which may have been due to the destruction by ultrasonic effects (including cavitation and mechanical effects) of intermolecular and intramolecular hydrogen bonds maintaining the triple-helix structure [44]. At 20/40 kHz and 20/60 kHz, the red shift increased with a relatively weak trend, which may have been due to the transformation of the ordered triple-helix structure of polysaccharides into a relatively loose triple-helix structure or due to partial destruction under the ultrasonic action at these frequencies. When the ultrasonic frequency was 20 kHz and 40 kHz, the red shift was like that of the original lentinan, suggesting that, under these ultrasound treatments, the lentinan still maintained a relatively orderly triple-helix structure.

#### 3.2.2. Molecular Weight and Chain Conformation Analysis

The molecular and conformational parameters of lentinan were determined by SEC-MALLS. As shown in Figure 3B, the chromatographic peak of original lentinan presented a single and nearly normal distribution, indicating a polysaccharide with high homogeneity. However, after ultrasonic treatment, the chromatogram of lentinan showed an obvious shoulder peak, especially at the frequencies of 60 kHz, 40/60 kHz, and 20/40/60 kHz, indicating that the molecular weight of lentinan changed from uniform to uneven. In addition, Table 2 shows that the molecular weight of lentinan significantly decreased after ultrasonic treatment with different frequencies, with the greatest decrease observed for 20/40/60 kHz, followed by 40/60 kHz and 60 kHz, indicating that the superposition of ultrasonic frequencies enhanced the cavitation effect, aggravated the breaking of the lentinan glycoside chain, and further led to a reduction in its molecular weight. The polydispersity coefficient (M_w_/M_n_) represents the molecular weight dispersion degree of polysaccharides. A smaller dispersion coefficient (M_w_/M_n_) indicates a more uniform molecular weight distribution [45]. After ultrasonic action of 60 kHz and 20/40/60 kHz, the polydispersity coefficient (M_w_/M_n_) increased, indicating that the molecular weight changed from homogeneous to uneven, consistent with the distribution of chromatographic peaks shown in Figure 2B. The results indicated that the intramolecular and intermolecular hydrogen bonds that maintain the triple-helix chain were broken by ultrasound, thus transforming into a single-strand compliant chain [46].

The chain conformation of polysaccharides can be determined by the exponent α, i.e., the relationship between <S^2^>_z_^1/2^ and M_w_, which is expressed by the relation <S^2^>_z_^1/2^ = KM_w_^α^. Usually, when α is about 0.3, the macromolecule is curled into a sphere. The exponent α is 0.5–0.6 for flexible chains and more than 0.6 for stiff or wormlike polymers in a good solvent [47]. As shown in Figure 3C, the values of α decreased to varying degrees due to various ultrasonic frequency modes, among which 20/40/60 kHz exhibited the lowest value (0.605), followed by 40/60 kHz (0.633) and 60 kHz (0.746), consistent with the changes in lentinan M_w_ after ultrasonic treatment. These results indicated that the original lentinan existed in the form of a rigid chain in aqueous solution; then, under the effect of ultrasound, the stiffness of lentinan chain decreased and gradually changed to a flexible chain. This may be because ultrasonic shear forces and cavitation effects broke the hydrogen bonds holding the rigid chains together, making them supple.

The structure sensitivity factor ρ (ρ = R_g_/R_h_) was used to evaluate the flexibility and rigidity of polysaccharide molecular chains, and the general rules are as follows: when ρ is equal to 0–0.77, the product is in a hard sphere conformation; when ρ = 1.0−1.1, the product is a highly branched chain; when ρ = 1.5−1.8, the product is a linear compliant chain; when ρ > 2, the product is a rigid chain. As shown in Table 2, the values of ρ decreased with the increase in ultrasonic frequency, among which 20/40/60 kHz exhibited the lowest value, followed by 40/60 kHz and 60 kHz. With the action of ultrasound, the ρ values were all between 1.42 and 1.93, indicating that the polysaccharide chain changed from a rigid chain to a flexible chain.

The morphology of polysaccharide molecular chains can be evaluated by the fractal dimension d_f_ value (d_f_ =1/α; 1 for a rigid rod chain; 5/3–2 for a linear random group; 3 for a three-dimensional uniform sphere). As shown in Table 2, the original lentinan had a d_f_ value of 1.08, indicating a rigid chain conformation. However, under the ultrasonic action of 40/60 kHz and 20/40/60 kHz, the d_f_ values increased to 1.58 and 1.60, respectively, indicating that lentinan at this frequency was linear and irregular, consistent with the above results.

#### 3.2.3. Intrinsic Viscosity Measurement

As an important molecular parameter, [η] is frequently used to study molecular size, chain stiffness, and solvent properties of polysaccharides in dilute solutions. In general, a smaller value of [η] indicates that the polysaccharide chain is a relatively tight coil, while a larger value of [η] indicates that the polysaccharide chain is a relatively expanded rigid chain [48]. As shown in Figure 3D, the original lentinan had the highest [η] value (155.82), indicating that lentinan without ultrasonic treatment was a relatively extended and ordered rigid chain. After different ultrasonic treatments, the values of [η] decreased. In single-frequency ultrasonic mode, as the frequency increased, the [η] value decreased from 144.54 to 59.17, indicating that the increase in ultrasonic frequency enhanced the cavitation effect and aggravated the destruction of polysaccharide chains. Under dual-frequency conditions, the most obvious change occurred at 40/60 kHz (decreased to 52.36), whereas the values at both 20/40 kHz and 20/60 kHz were higher than that at 60 kHz, which could be explained by the possible interference of ultrasound with different frequencies at certain propagation locations or phases. This may have caused an elimination phenomenon, thereby reducing the cavitation intensity, and resulting in a corresponding reduction in ultrasonic degradation. Under the action of 20/40/60 kHz ultrasound, the value of [η] decreased the most, which may be because the superposition of ultrasound frequencies enhanced the effect of ultrasound (shear force, cavitation effect, etc.), resulting in the chain scission and significant changes in chain conformation.

#### 3.2.4. XRD Analysis

**Figure 4:**
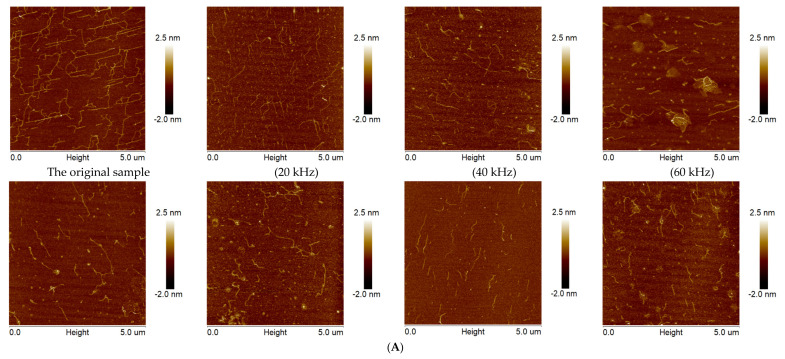

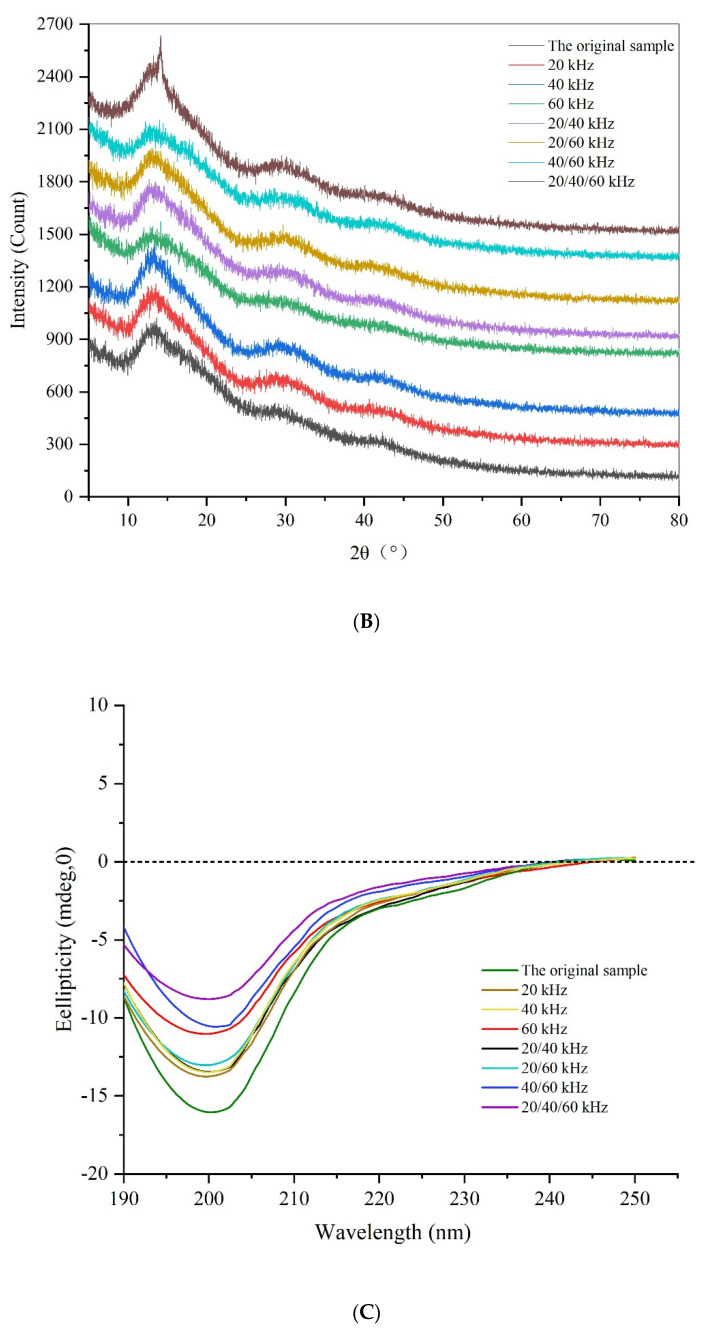
Morphology of lentinan before and after different frequency and combined ultrasound treatment according to atomic force microscopy (**A**), X−ray diffraction spectra (**B**), and CD analysis (**C**).

XRD analysis is often used to investigate the crystalline structure of samples. The XRD analysis results of lentinan before and after ultrasonic treatment at different frequencies are exhibited in Figure 4B. It can be noted that the lentinan without ultrasonic treatment had a broad absorption peak at 2θ = 14°, indicating that the crystallinity of original lentinan was low [49]. However, after treatment with 60 kHz and 40–60 kHz ultrasound, the diffraction peak of lentinan became flat at about 2θ = 14°, which may have been due to the destruction of intramolecular and intermolecular hydrogen bonds of lentinan, resulting in varying degrees of changes in the crystal structure of lentinan [50]. In particular, it should be noted that, at 20/40/60 kHz, the diffraction peak of lentinan suddenly became high and discontinuous at about 2θ = 14°, which may have been because the ordered structure of lentinan was destroyed by ultrasonic at this frequency, making it amorphous.

#### 3.2.5. CD Analysis

Due to the sensitivity of CD to molecular conformation, CD was commonly used for studying the transformation of polysaccharide chain conformations in aqueous solutions in recent years [20]. As shown in Figure 4C, lentinan presented a negative Cotton effect before and after different frequencies and combinations of ultrasound. The maximum negative peak of lentinan was found at 202 nm, and the ellipticity was about −16.5°·cm^2^·g^−1^. The position of the negative peak of lentinan changed slightly after ultrasonic treatment, especially at 60 kHz and 20/40/60 kHz, shifting from 202 nm to 199 nm, which may have been due to the n–π* transition of the carboxyl group. The intermolecular and intramolecular interactions affect the optical rotation of the carboxyl chromophore. Studies have shown that the changes in the peak position of negative Cotton may be due to the interaction between polysaccharide molecular chains, resulting in a change in chain conformation in solution. In addition, the ovality of lentinan changed obviously under the same ultrasonic treatment. At the frequencies of 60 kHz, 40/60 kHz, and 20/40/60 kHz, the ellipticity of lentinan was −11.6°·cm^2^·g^−1^, −10.3°·cm^2^·g^−1^ and −8.4°·cm^2^·g^−1^, respectively, indicating that the molecular asymmetry changed [51]. The intermolecular and intramolecular hydrogen bonds of lentinan were broken at different frequencies, and the chain conformation of lentinan in solution was changed. The conformation of lentinan in solution could change the ellipticity and peak position of CD. In conclusion, different frequency modes of ultrasound, especially 60 kHz, 40/60 kHz, and 20/40/60 kHz, could cause the rigid triple-strand helical chain of lentinan to gradually break and melt, transforming into a flexible single helical chain. This is consistent with other experimental results.

#### 3.2.6. AFM Analysis

To provide direct evidence for the chain conformation of lentinan under different frequency ultrasound, AFM technology was used to observe and evaluate the surface morphology [52]. As shown in Figure 4A, the structure of original lentinan was dominated by long rigid and straight chains, and the polysaccharide chains were arranged orderly, accompanied by multiple flexible branches. However, after treatment using ultrasound with different frequencies, the arrangement of lentinan chains became disordered, the branched chains became soft, short, and low polysaccharide chains, and some polysaccharide chains were mutually wound to form random coils. These changes were significantly affected by the frequency modes of ultrasound, and the effect of the triple-frequency mode was much greater than that of single- and dual-frequency ultrasound. This may have been because the superposition of ultrasonic frequencies enhanced the cavitation effect, which destroyed the intramolecular and intermolecular hydrogen bonds maintaining the triple-helical structure of the polysaccharide chain, resulting in the gradual fracture of polysaccharide branch side-chains [15], supporting the rigidity of polysaccharide molecules and limiting the formation of aggregation, tangles, and bonding zones [20]. The triple-helix structure gradually became loose before finally dissociating into a single-strand short-chain structure with side-chains. This result is consistent with the conclusion that the triple-helix structure disappeared under this condition in the Congo red test. 

### 3.3. Immunomodulatory Activities

In recent years, natural polysaccharides have attracted extensive attention because of their nontoxicity and strong immunomodulatory activity. As an immune modulator, lentinan can regulate the immune response by stimulating cell proliferation, maturation, and differentiation, promoting cytokine release, and activating intracellular signaling pathways [53]. However, as mentioned above, the spatial conformation of lentinan changed significantly after ultrasonic treatment with different frequency modes, and the effects of these changes on its immunomodulatory activity still need to be further studied. Macrophages can phagocytize deformable cells, attack invaders, and respond sensitively to infection, tumor, and inflammation; thus, they are often used as cell models to evaluate immune activity. When polysaccharides bind to the receptors on the surface of macrophages, these receptors can trigger different signal pathways to activate macrophages, promote macrophages to kill pathogens by directly phagocytizing antigens, trigger an immune response, induce intracellular iNOS to release NO (inflammatory mediator), and promote cells to secrete cytokines including tumor necrosis factor (TNF-α) and interleukin factors (IL-1, IL-6, IL-10) [54]. Therefore, in this study, mouse monocyte macrophages RAW264.7 were used to explore the immunomodulatory effect of lentinan by detecting the proliferation, phagocytic index, and the ability to secrete NO and cytokines of lentinan on macrophages, thereby providing basic data for the in-depth study of lentinan’s immune activity.

#### 3.3.1. Effects of Lentinan on Cell Viability

**Figure 5:**
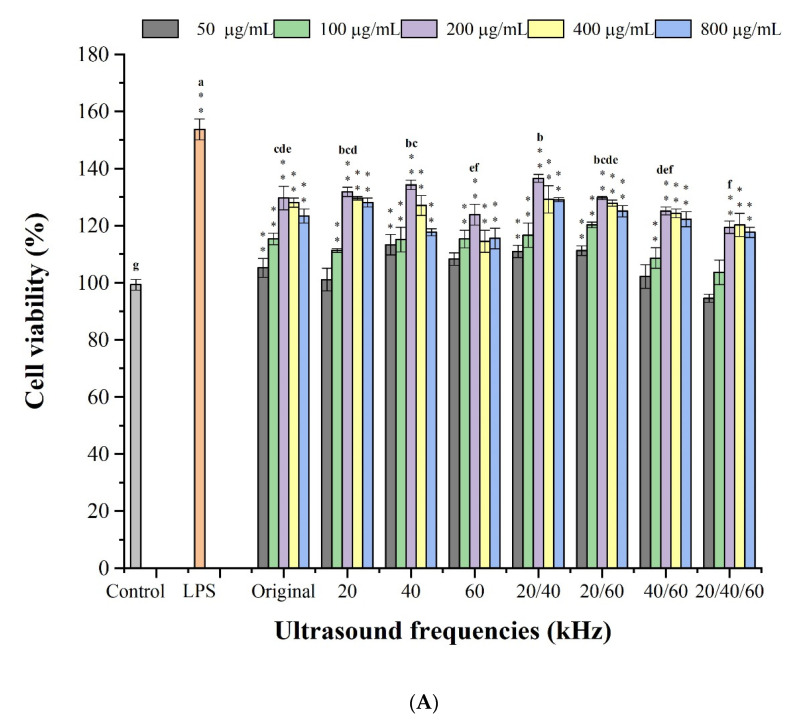

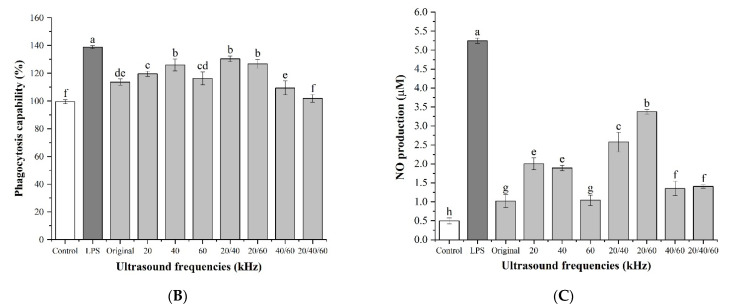
Immunomodulatory activities: effect of lentinan on cell viability (**A**); phagocytic activities (**B**); NO production (**C**). ** Significant correlation at the 0.01 level. Different letters in the figure indicate significant differences (*p* < 0.05).

The effects of lentinan on the viability of RAW264.7 cells were determined using the CCK-8 assay. As shown in Figure 5A, no cytotoxicity was found in the range of test concentrations (50–800 μg/mL); in the range of 50–200 μg/mL, all lentinans stimulated the proliferation of RAW264.7 cells in a dose-dependent manner. It is noteworthy that 200 μg/mL lentinan had the highest proliferation effect on RAW264.7 cells. Therefore, 200 μg/mL was selected for the subsequent analysis. Accordingly, the influence of 200 μg/mL lentinan of each component on macrophage proliferation was compared; the influence of lentinan on RAW264.7 proliferation was the most significant at 40 kHz and 20/40 kHz, while the influence of lentinan at 60 kHz, 40–60 kHz, and 20/40/60 kHz on cell proliferation was reduced compared to that without ultrasonic treatment.

#### 3.3.2. Effects of Lentinan on Cell Phagocytic Activity

Activated macrophages play a crucial role in the innate immune response and tissue repair, which can kill pathogens directly through phagocytosis. The activation of macrophages can be assessed by the increase in phagocytic activity, and the influence of lentinan on the phagocytic activity of RAW264.7 cells could be reflected by a neutral red test. As shown in Figure 5B, except for the frequency of 20/40/60 kHz, other components of lentinan all had the capacity (*p* < 0.01) to promote the phagocytosis of RAW264.7 cells. Notably, at 20/40 kHz, the macrophage phagocytic activity reached the highest value and approached the LPS group.

#### 3.3.3. Effects of Lentinan on Cell Morphology

**Figure 6:**
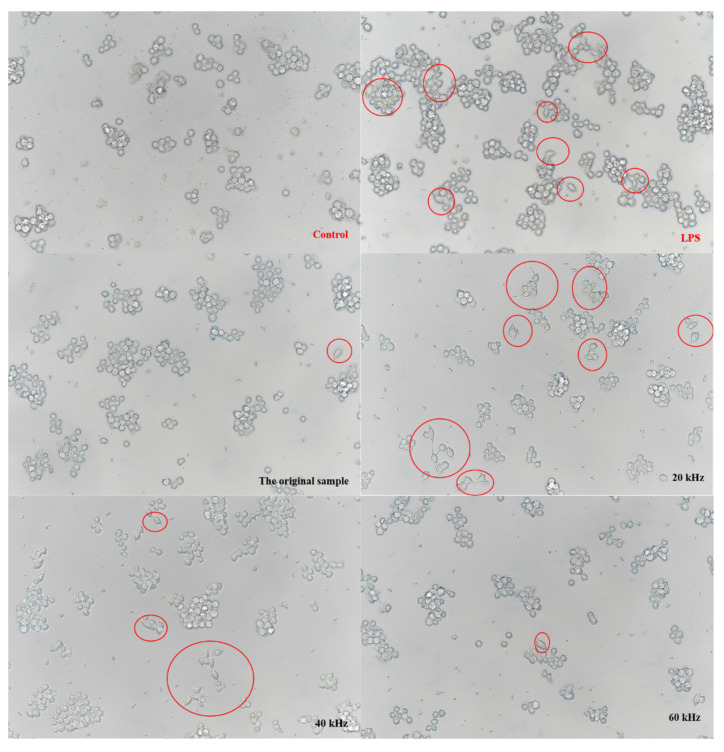

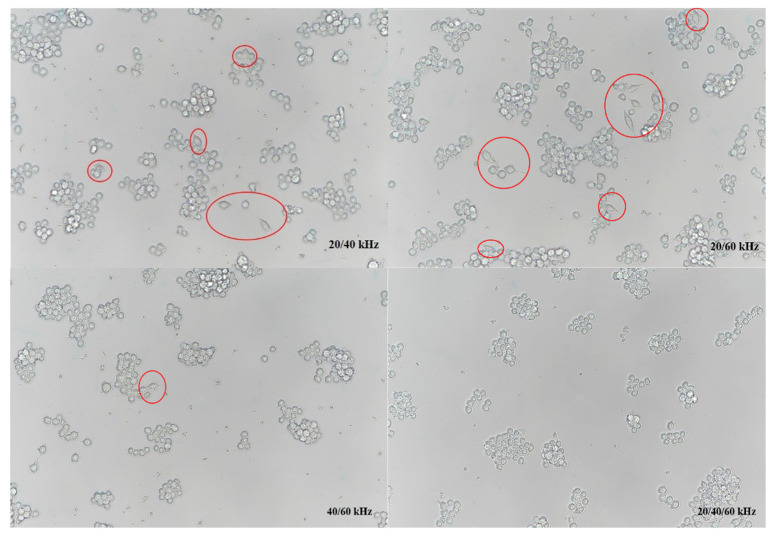
The effect of lentinan on the morphological features of RAW264.7 macrophages.

The deformation of macrophages, especially the spindle morphology, generally indicates the activation of macrophages. As shown in Figure 6, the control inactive cells were mostly round or oval with uniform size. In the sample groups, the cells changed from round to polygons or long spindles, with a pseudopod appearance, exhibiting typical activated morphology. The sample group had the most pronounced cell changes at 20/40 kHz and 20–60 kHz. The results are similar to those obtained in the phagocytosis experiments.

#### 3.3.4. Effect of Lentinan on Cell NO Production

An activated macrophage can generate a plurality of immune response reactions and release NO as a gas signal molecule, so as to promote the induction or inhibition of macrophage apoptosis, thereby playing an immune regulation effect [55]. Therefore, NO can serve as a quantitative indicator of macrophage activation. As shown in Figure 5C, the NO generation amount (0.51 μM) of the control group was far lower than that of the other lentinans except for those treated with 60 kHz ultrasound (*p* < 0.01), suggesting that ultrasonic action could promote the activation of macrophages. Notably, the differences between the different components were also large, with the 20/60 kHz component having the highest NO generation (2.68 μΜ) and the 60 kHz component having the lowest NO generation (1.10 μΜ).

#### 3.3.5. Effect of Lentinan on Cell Cytokine Secretion

**Figure 7:**
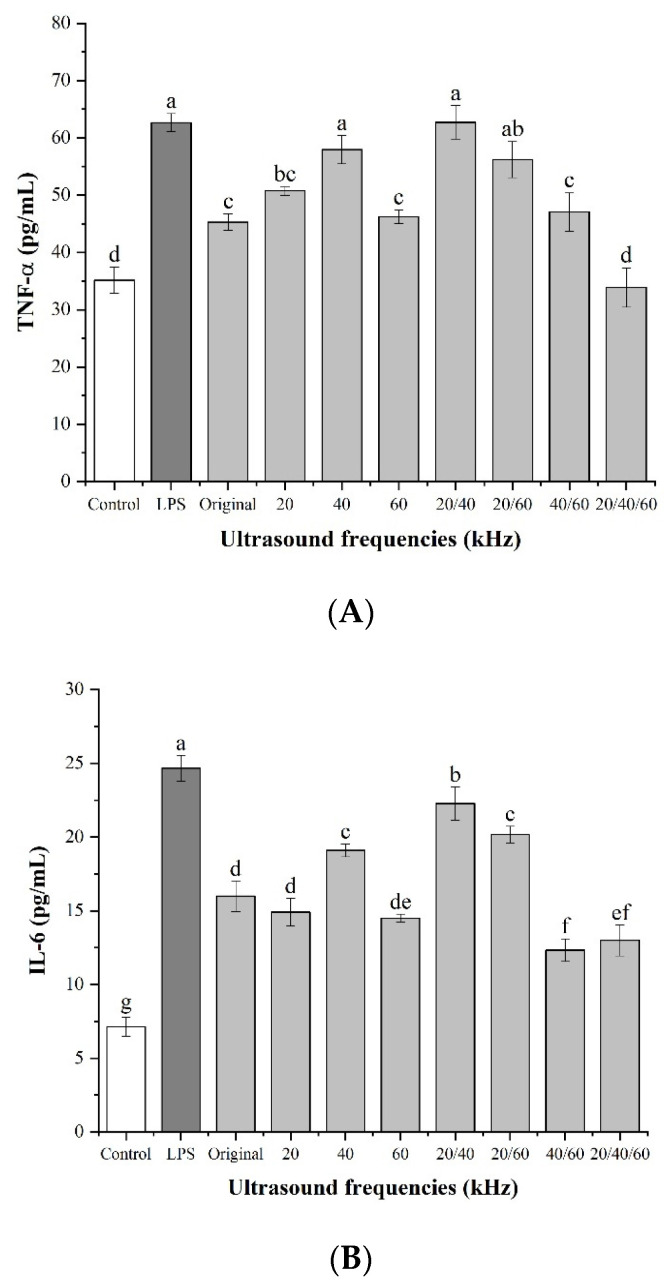

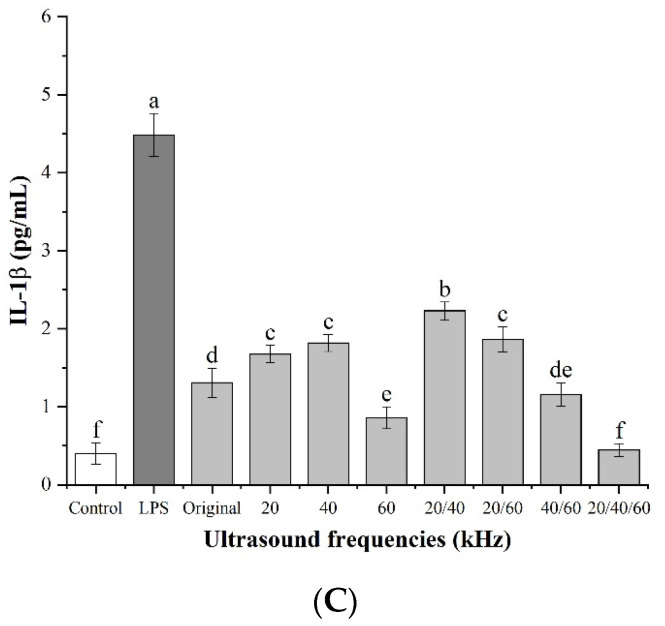
Cytokine secretion: (**A**) TNF-⍺; (**B**) IL-6; (**C**) IL-1β. Different lowercase letters denote a statistically significant difference at *p* < 0.05 and *p* < 0.01 with the control group.

Polysaccharides can indirectly kill tumors by activating macrophages to secrete inflammatory factors, such as TNF-α, IL-6, and IL-1β, and exert an immune regulation effect. As shown in Figure 7A–C, lentinans treated with different frequency modes significantly promoted the secretion of TNF-α, IL-6, and IL-1β compared with the control (ρ < 0.01). Especially at 20/40 kHz, the production levels of the three immune factors were only second to those of LPS. However, at 60 kHz and 20/40/60 kHz, the release amount of the three immune factors was significantly lower than that at the other frequency modes, which may have been caused by the changes in the chemical structure and chain conformation of lentinan after ultrasonic treatment. 

### 3.4. Correlation Analysis

In order to evaluate the structure–activity relationship of lentinan more clearly, a Pearson correlation analysis between chain conformation parameters and immunoreactivity parameters was carried out. The results are shown in Table 3. 

The amount of NO and IL-1β secretion in RAW267.4 cells was shown to be not significantly correlated with chain conformational parameters α, ρ, and d_f_ (two-tailed *p* > 0.05). However, only the secretion of TNF-α and IL-6 was negatively correlated with the conformation d_f_ (two-tailed *p* < 0.05). There was no significant correlation between the secretion of TNF-α and IL-6 and other chain conformation parameters α and ρ, but the two-tailed values were all less than 0.1 and close to 0.05. Pearson correlation coefficients were all between 0.6 and 0.8, showing moderate correlation. The results indicated that the chain conformation of lentinan affected its immunological activity to a certain extent.

Some studies have found that polysaccharides, as exogenous biological macromolecules, can be recognized by the pattern recognition receptors of natural immune cells before entering the body to play an immune-regulatory role. Several pattern recognition receptors of polysaccharides have been reported, including Dectin-1, Toll receptor, complement receptor 3, and scavenger receptor. The monosaccharide composition, molecular weight, glycosidic bond, branching degree, and triple-helix conformation of polysaccharides played a profound role in binding to receptors. Zheng et al. [56] found that both the spherical and rigid chain conformation of water-soluble yeast β-glucan could interact with Dectin-1, but the spherical conformation had stronger interactions than the rigid chain conformation. Adams et al. [57] reported that the side-chain structure of β-(1,3)-d-glucan was important in its interaction with Dectin-1. Notably, Chao et al. [58] found that the triple-helix conformation of polysaccharides exerted immune-stimulating activity by promoting the production of TNF-α in macrophages. In this study, Congo red, atomic force microscopy, and a series of solution chain conformation theories were used to observe the conformation changes of lentinan chains under different ultrasound frequency modes. In particular, at 60 kHz and 20/40/60 kHz, lentinan transformed from a rigid triple-helix chain to a flexible single-helix chain, and the side-chain broke, resulting in the lowest levels of immune factors and the worst immunocompetence. On the other hand, at 20/60 kHz, 20/40 kHz, and 40 kHz, the polysaccharide remained in a triple-helical conformation, but its rigid chains gradually transitioned to looser, flexible chains, and the side-chains became soft. Therefore, these findings suggest that the triple-helix conformation plays an important role in lentinan immunocompetence. In the presence of a triple-helix chain, the flexible loose chain had better immunocompetence than the rigid tight chain. This may have been due to the soft loose chains binding more readily to specific sites on the surface of the receptor. Therefore, future studies can investigate the immune mechanism of polysaccharides with different helical chain conformations and rigid flexible chains as a function of the affected receptors and pathways.

## 4. Conclusions

In this study, the effects of ultrasound with different frequency modes on the chemical structure, chain conformation, and immune activity of lentinan were studied, and the structure-activity relationship of lentinan was further discussed. The results showed that, under different modes of ultrasound, the monosaccharide composition and functional groups of lentinan did not change significantly, but its molecular weight and particle size decreased significantly. The polysaccharide chain changed from a rigid triple-helix chain to flexible single helix chain, while the side-chain broke significantly, which may have been the result of intermolecular and intramolecular hydrogen bonds. In addition, the immune activity of lentinan with a triple-helix conformation showed stronger immune activity, and the flexible chain polysaccharide was more active than the rigid chain polysaccharide. In summary, the effect of multifrequency ultrasound on the conformation of lentinan chain was more significant than that of single-frequency ultrasound, and the conformation of the lentinan chain played an important role in enhancing the immune activity of lentinan. This may have important theoretical value and practical significance for guiding the application of different ultrasound frequency modes in the processing of carbohydrate foods. However, the interaction between polysaccharides with different conformations and different receptors remains unclear. Therefore, the interaction between lentinan with different chain conformations after ultrasonic treatment and cell membrane receptors will be further studied to explore the relationship between polysaccharide chain conformation and immune molecular mechanism. 

## Figures and Tables

**Table 1:** Monosaccharide composition of lentinan under different ultrasonic frequency modes before and after ultrasonic treatment.

Samples		Monosaccharide (mol.%)		
	Ara	Man	Glu	Gal
Original	1.57 ± 0.02	10.42 ± 0.17	77.51 ± 0.73	10.50 ± 0.35
20 kHz	1.69 ± 0.03	10.79 ± 0.12	78.53 ± 0.99	9.09 ± 0.13
40 kHz	1.73 ± 0.03	9.40 ± 0.11	79.37 ± 1.35	9.50 ± 0.25
60 kHz	1.81 ± 0.05	8.11 ± 0.05	81.01 ± 1.21	9.07 ± 0.19
20/40 kHz	1.68 ± 0.04	8.96 ± 0.07	79.49 ± 0.87	9.87 ± 0.11
20/60 kHz	1.67 ± 0.04	8.32 ± 0.09	80.79 ± 1.03	9.22 ± 0.12
40/60 kHz	1.89 ± 0.05	8.52 ± 0.06	81.33 ± 1.29	8.26 ± 0.06
20/40/60 kHz	1.83 ± 0.03	8.20 ± 0.04	81.53 ± 1.05	8.44 ± 0.21

**Table 2:** Physicochemical properties and molecular parameters of lentinan at different ultrasonic frequency modes before and after ultrasonic treatment.

Samples	M_n_ (g/mol)	M_w_ (g/mol)	M_w_/M_n_	<S^2^>_z_^1/2^ (nm)	R_h_ (nm)	α ^a^	ρ ^b^	d_f_^c^	Conformation
Original	2.218 ± 0.353 ×$\;10^{6}$	2.804 ± 0.191 ×$\;10^{6}$	1.264 ± 0.051	88.45 ± 3.22	40.79 ± 2.37	0.923	2.17	1.08	Rigid chain
20 kHz	1.731 ± 0.105 ×$\;10^{6}$	1.336 ± 0.112 ×$\;10^{6}$	1.295 ± 0.013	60.16 ± 3.24	31.24 ± 1.09	0.825	1.93	1.21	Rigid chain
40 kHz	1.103 ± 0.195 ×$\;10^{6}$	1.265 ± 0.181 ×$\;10^{6}$	1.146 ± 0.022	55.67 ± 3.31	29.18 ± 2.03	0.814	1.91	1.23	Rigid chain
60 kHz	6.008 ± 0.445 ×$\;10^{5}$	8.693 ± 0.597 ×$\;10^{5}$	1.446 ± 0.019	42.13 ± 1.99	25.87 ± 2.23	0.746	1.63	1.34	Linear flexible chain
20/40 kHz	8.004 ± 0.375 ×$\;10^{5}$	9.399 ± 0.461 ×$\;10^{5}$	1.174 ± 0.037	48.72 ± 2.07	25.65 ± 2.05	0.802	1.90	1.25	Semirigid chain
20/60 kHz	7.582 ± 0.423 ×$\;10^{5}$	8.281 ± 0.315 ×$\;10^{5}$	1.092 ± 0.010	45.36 ± 1.64	24.15 ± 2.04	0.819	1.88	1.22	Semirigid chain
40/60 kHz	6.052 ± 0.615 ×$\;10^{5}$	7.760 ± 0.237 ×$\;10^{5}$	1.282 ± 0.041	28.17 ± 1.17	18.61 ± 1.51	0.633	1.51	1.58	Random coil
20/40/60 kHz	4.102 ± 0.409 ×$\;10^{5}$	6.607 ± 0.365 ×$\;10^{5}$	1.607 ± 0.041	25.15 ± 1.35	17.17 ± 1.16	0.605	1.42	1.65	Random coil

^a^ The exponent of power law functions <S^2^>_z_^1/2^ = KM_w_^α^. ^b^ The structural parameter ρ = <S^2^>_z_^1/2^/R_h_. ^c^ The fractal dimension d_f_ = 1/α.

**Table 3:** Pearson correlation analysis between chain conformation parameters and immunoreactivity parameters.

		α	ρ	d_f_
NO	Pearson correlation	0.315	0.329	−0.385
	Sig. (2-tailed)	0.447	0.426	0.347
TNF-α	Pearson correlation	0.661	0.648	−0.721 *
	Sig. (2-tailed)	0.074	0.082	0.044
IL-6	Pearson correlation	0.661	0.681	−0.723 *
	Sig. (2-tailed)	0.065	0.063	0.043
IL-1β	Pearson correlation	0.550	0.614	−0.586
	Sig. (2-tailed)	0.158	0.105	0.127

* Significant correlation at the 0.05 level (two-tailed).

## Data Availability

The datasets used and analyzed are available from the authors.

## Supplementary Materials

The following supporting information can be downloaded at: https://www.mdpi.com/article/10.3390/foods11162470/s1. Figure S1: multi-frequency power ultrasound (A); Schematic diagram of the ultrasound device (a: ultrasonic transducer; b: ultrasonic generator; c: flat divergent ultrasound angle) (B); Waveform diagram of different frequency mode ultrasonic device (From T0 to T0+T is one ultrasonic cycle) (C).
