# Supplementary material for: Effects of Ultrasound Modification with Different Frequency Modes on the Structure, Chain Conformation, and Immune Activity of Polysaccharides from Lentinus edodes
Source: Foods. 2022 Aug 16;11(16):2470. doi: 10.3390/foods11162470 (PMC9407330; doi:10.3390/foods11162470)
Supplement: Supplementary file 1 [file foods-11-02470-s001.zip › foods-1859533-supplementary.pdf]

Supplementary Material for the Manuscript Entitled:

Effects of Ultrasound Modification with Different Frequency Modes on the Structure, Chain Conformation, and Immune Activity of Polysaccharides from *Lentinus edodes*

(A)

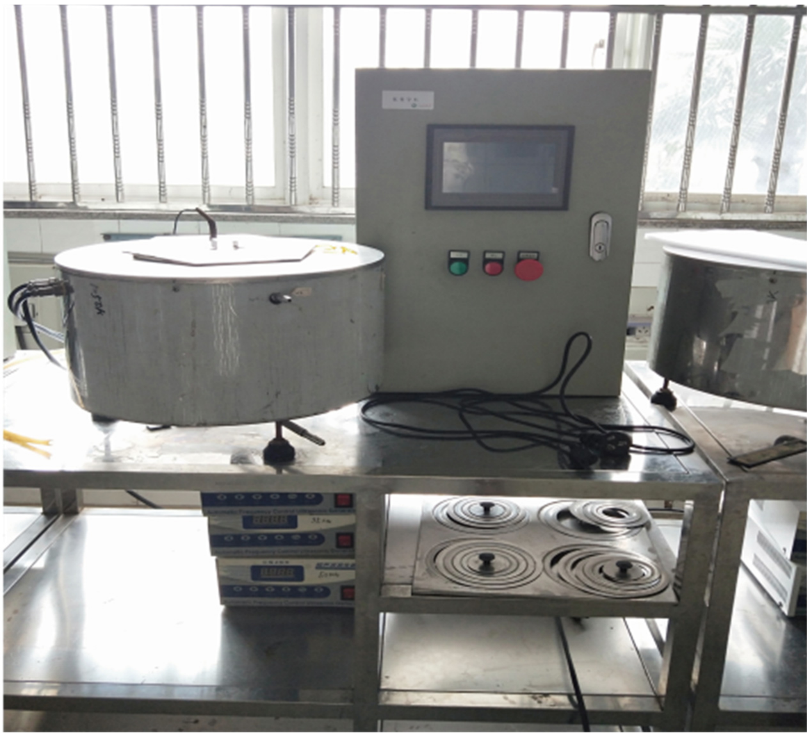

(B)

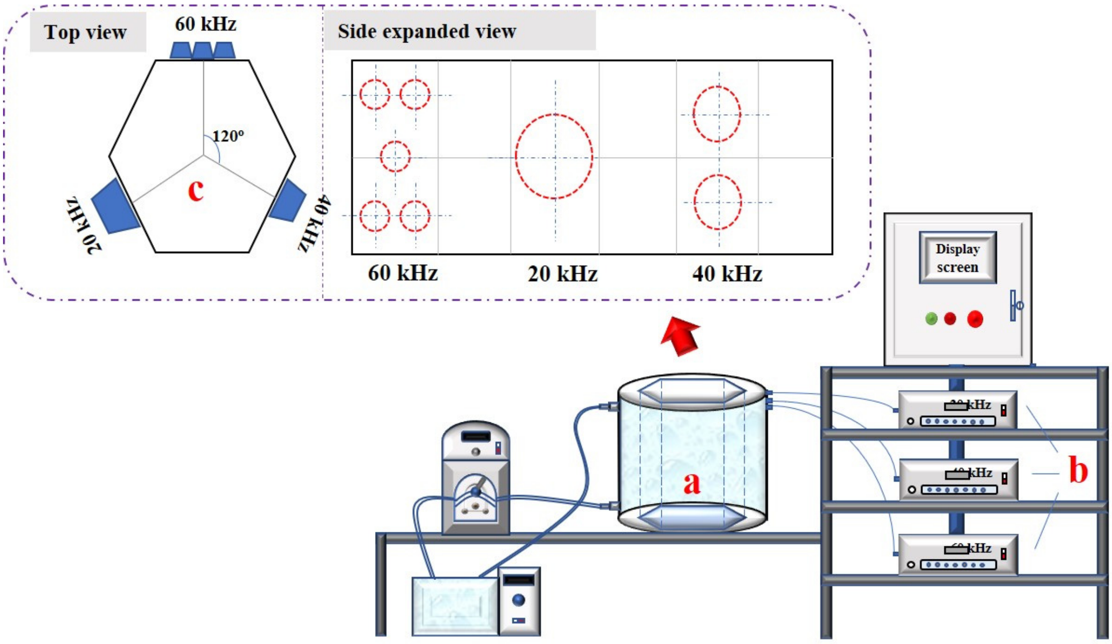

Schematic diagram of Multi-frequency Power Ultrasound (MFPU)

(C)

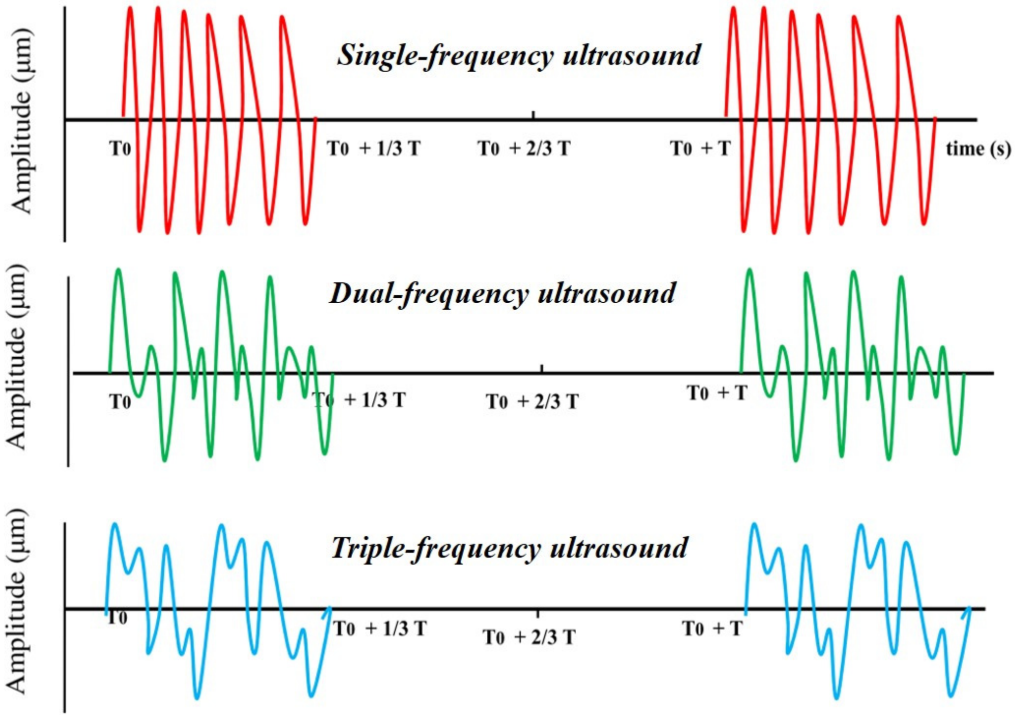

Figure S1. multi-frequency power ultrasound (A); Schematic diagram of the ultrasound device (a: ultrasonic transducer; b: ultrasonic generator; c: flat divergent ultrasound angle) (B); Waveform diagram of different frequency mode ultrasonic device (From T_0 to T_0+T is one ultrasonic cycle) (C).
